# The characteristics and influence of iatrogenic fracture comminution following antegrade interlocking nailing for simple femoral shaft fractures, a retrospective cohort study

**DOI:** 10.1186/s12891-022-05418-2

**Published:** 2022-05-14

**Authors:** Jou-Hua Wang, Hao-Chun Chuang, Wei-Ren Su, Wei-Lun Chang, Fa-Chuan Kuan, Chih-Kai Hong, Kai-Lan Hsu

**Affiliations:** 1 Department of Orthopaedic Surgery, College of Medicine, National Cheng Kung University Hospital, National Cheng Kung University, Tainan, Taiwan; 2 Department of Biomedical Engineering, National Cheng Kung University, Tainan, Taiwan; 3 Division of Orthopaedics, Department of Surgery, National Cheng Kung University Hospital DouLiou Branch, National Cheng Kung University, Douliu, Yunlin Taiwan; 4 Department of Orthopaedics, National Cheng Kung University Hospital, No.138, Sheng Li Road, 704 Tainan, Taiwan

**Keywords:** Femoral shaft fracture, Iatrogenic fracture comminution, Incidence, Nonunion

## Abstract

**Aim:**

The incidence and characteristics of iatrogenic comminution (IC) are unknown, and the influence of IC on fracture union is unclear. This study was aimed to investigate the (1) incidence and characteristics of IC and (2) the outcomes of IC following antegrade interlocking nailing of simple femoral shaft fractures.

**Methods:**

We retrospectively collected data on patients who experienced simple femoral shaft fractures and underwent antegrade interlocking nailing between February 2009 and December 2016. The incidence and characteristics of IC were examined. According to the presence of IC, patients were divided into two groups: an IC group and a non-IC (NIC) group. Demographic information and nonunion rates were compared between the two groups. Potential risk factors for IC (age, gender, body mass index (BMI), nail fit ratio, reduction technique, and greater trochanter nail entry) were analyzed using univariate and multivariate logistic regression. The aforementioned variables, along with IC occurrence, were also assessed as potential risk factors for nonunion at 12 and 24 months after operation using multivariate logistic regression.

**Results:**

Of the 211 total patients, IC occurred in 20.9% (*n* = 44) of patients. Most ICs were found at the level of the isthmus, and involved the medial cortex. Compared with the NIC group, higher nonunion rates were observed in the IC group at 12 months (31.8% vs. 12.5%, *p* = 0.002) and 24 months (18% vs. 6.5%, *p* = 0.017) after surgery. Age older than 35 years old was related with the occurrence of IC in univariate analysis. Multivariate analysis found no risk factor associated with IC. Open reduction technique, IC occurrence and higher BMI were identified as the risk factors of nonunion at 12 months and 24 months after surgery in multivariate analysis.

**Conclusion:**

IC is a non-rare complication in antegrade interlocking nailing of simple femoral shaft fractures and was associated with higher nonunion rate. Age older than 35 years old showed a trend toward increasing risk of iatrogenic fracture comminution. In multivariate analysis, open reduction technique, IC occurrence and higher BMI significantly correlated with fracture nonunion.

**Level of evidence:**

Level IV.

## Introduction

Intramedullary nailing (IMN), a common orthopedic surgical procedure, is currently the standard treatment for femoral shaft fractures in adults [1]. Research demonstrated that the percutaneous application of IMN resulted in high union rates and favorable functional outcomes [2]. Despite these advantages, complications such as mechanical failure, infection, delayed union, malunion, or nonunion can develop [1, 3]. Iatrogenic complications, including fracture comminution and ipsilateral femoral neck fractures have also been reported in the literature [3–6].

Iatrogenic fracture comminution may interfere with fracture reduction and can therefore make surgery more challenging. However, studies on the incidence, risk factors, and influence of iatrogenic comminution (IC) are lacking. Hence, we retrospectively reviewed a cohort of patients with femoral shaft fractures treated at our hospital. The purposes of this study were to investigate (1) the incidence and characteristics of IC and (2) the outcomes of IC following antegrade interlocking nailing of simple femoral shaft fractures.

## Materials and methods

### Study design and patient selection

This is a single-center, retrospective cohort trial conducted in a level I trauma center, and it was approved by the Institutional Review Board (B-ER-109-260). All methods were carried out in accordance with the Declaration of Helsinki. Potential participants with femoral fracture were identified using International Classification of Disease (ICD)-10 codes. All medical information for the identified patients was retrieved from their electronic medical records.

From February 2009 to December 2016, patients with simple femoral shaft fractures (Arbeitsgemeinschaft für Osteosynthesefragen and Orthopaedic Trauma Association (AO/OTA) classifications 32-A1, 32-A2, and 32-A3) who underwent antegrade interlocking nailing were included. The femoral shaft was defined between 5 cm distal to the lesser tuberosity and 9 cm proximal to the joint line [7]; any fractures that extended beyond this range were excluded. Patients were excluded if they received primary treatment with plate osteosynthesis or retrograde nailing or had delayed surgery for any reason. Patients with skeletal immaturity; open fractures classified as Gustilo III; wedge or multifragmentary fractures (AO/OTA 32-B or 32-C type); pathological fractures; or concomitant femoral neck, peri-trochanter, or condylar fractures were also excluded, along with patients lost to follow-up.

Eligible patients were divided into two groups: an IC group and a non-IC (NIC) group. IC was defined as any extension of the radiolucent line or fragment around the initial fracture that was not present before surgery. Iatrogenic fractures of the femoral neck were not considered as ICs.

### Collection of demographic data

The following demographic data were recorded: age, gender, fracture side (right or left), body mass index (BMI), fracture site relative to the isthmus (supraisthmic, isthmus, or infraisthmic), and width of the medullary canal. The following details of the surgery were also recorded: the patient’s posture (supine on the fracture table or lateral decubitus position), the reduction technique employed (open or closed reduction), nail entry point (via the greater trochanter or piriformis fossa), nail size, nail fit ratio (the ratio between nail size and the width of the medullary canal), and IC occurrence.

**Fig. 1:**
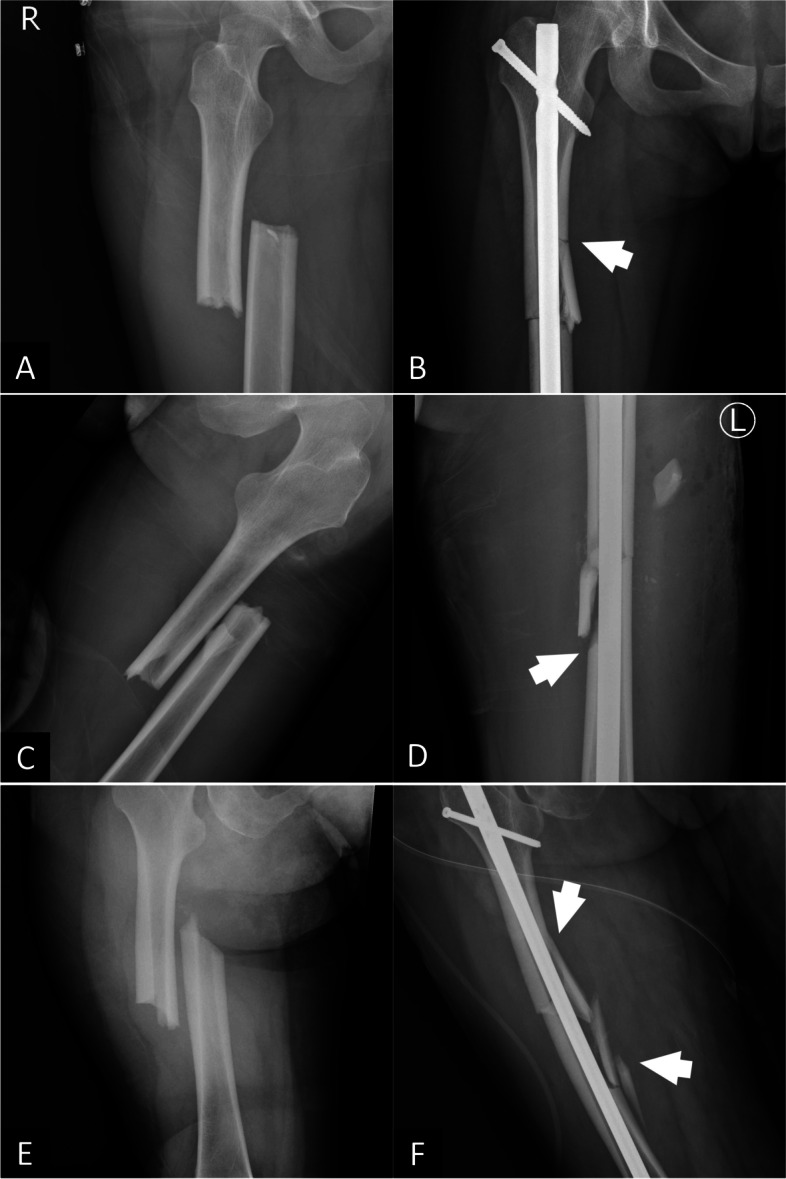
**A** A preoperative plain film of a transverse femoral shaft fracture, **B** a postoperative plain film with iatrogenic fracture comminution involving the medial cortex with proximal extension, **C** a preoperative plain film of a transverse femoral shaft fracture, **D** a postoperative plain film with iatrogenic fracture comminution involving the lateral cortex with distal extension, **E** a preoperative plain film of an oblique femoral shaft fracture, **F** a postoperative plain film showing iatrogenic fracture comminution involving the medial cortex with both proximal and distal extension

**Fig. 2:**
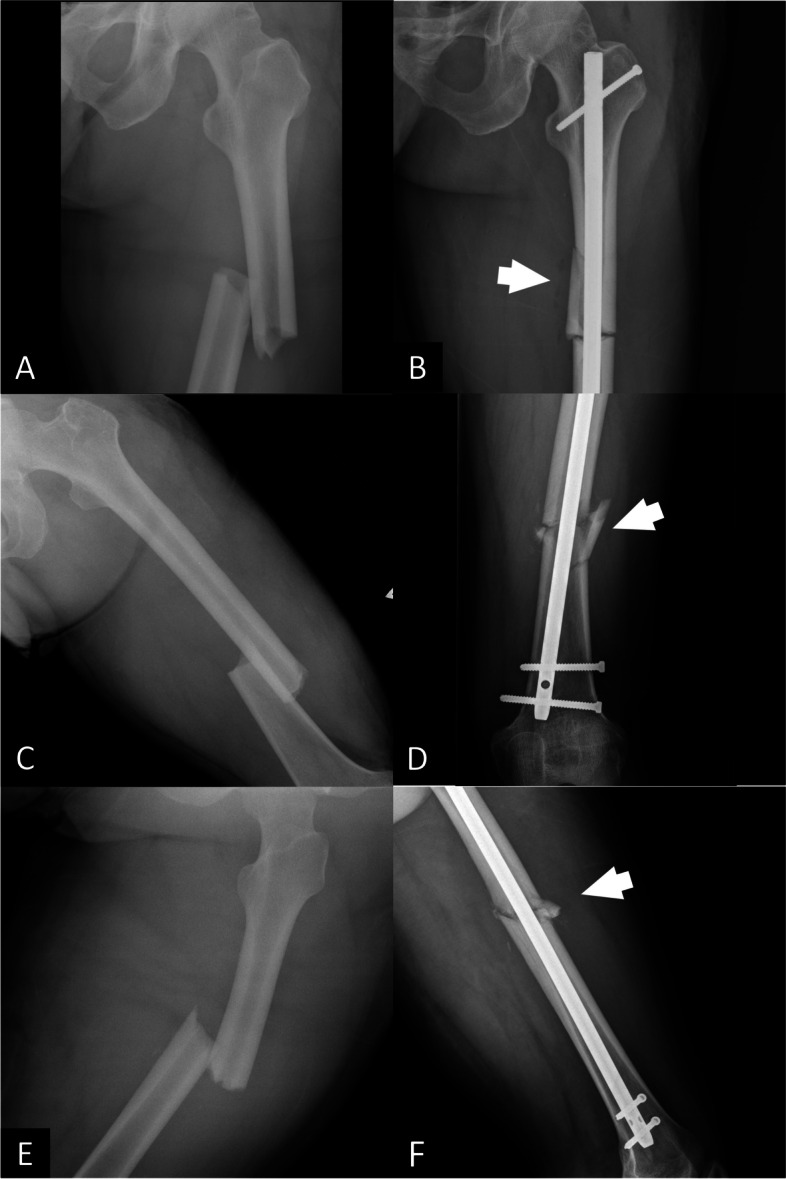
**A** A preoperative plain film of a transverse femoral shaft fracture, **B** a postoperative plain film showing an iatrogenic fracture comminution involving the medial cortex with proximal extension, **C** a preoperative plain film of a transverse femoral shaft fracture, **D** a postoperative plain film showing iatrogenic fracture comminution involving the lateral cortex with distal extension, **E** a preoperative plain film of a transverse femoral shaft fracture, **F** a postoperative plain film showing iatrogenic fracture comminution involving the anterior cortex with proximal extension

In the IC group, the characteristics of fracture comminution, including the location (supraisthmic, isthmus, or infraisthmic), the developed side (medial, lateral, anterior, posterior, long spiral, or multiple), and the direction of extension (toward the proximal direction, distal direction, or both) were also recorded, as illustrated in Figs. 1A to F and 2A to F.

### Treatment protocols

Surgery was performed within 24 h of injury. Systemic prophylactic antibiotics were administered intravenously once before surgery and continued for 1 day, with a maximum of three doses. Patients were in the supine position on the fracture table or in the lateral decubitus position depending on the surgeon’s preference. A standard antegrade technique was used for reamed IMN in all patients using the Zimmer Pressure Sentinel reamer (Zimmer USA, Warsaw, IN, USA) [8]. Two types of interlocking nails were used: the Aesculap Targon nail (Aesculap, Tuttlingen, Germany) and the Stryker T2 femoral nailing system (Stryker, Mahwah, NJ, USA). The smallest nail diameter available for both types of nails was 10 mm. Both types of nails were designed for piriformis fossa insertion.

The postoperative rehabilitation programs were similar for all participants. Partial weight bearing using crutches was allowed immediately after surgery except when IC was noted during the operation. Full weight bearing was allowed when some callus formation was noted on the plain film, which typically occurred 6 to 8 weeks after surgery.

The follow-up visits were planned during weeks 2, 4, and 6 and every 2 months thereafter for clinical and radiological evaluations. The minimum follow-up period was 24 months or until fracture union was confirmed both clinically and radiographically. All radiographs and medical charts were retrospectively reviewed independently by two orthopedic surgeons. Images of the patients were displayed and measured with digital imaging using medical image viewing software (πViewTM, INFINITT Co., Ltd., Seoul, South Korea).

### Outcome measures

The outcomes measured in our study were the characteristics of IC and their associations with nonunion. First, the incidence and features of IC were calculated and recorded in detail. Second, demographic data were compared between the IC group and the NIC group. Third, the nonunion rates in the IC group and NIC group were calculated and compared. Fracture healing was radiographically defined as three solid bridging callus ridges connecting the fracture fragment for both the anteroposterior (AP) view and the lateral view. Nonunion was diagnosed when fractures failed to reach the definition. In the IC group, fractures were divided into subgroups according to the side, location, and direction of the IC. Nonunion rates were compared between the subgroups.

### Statistical analysis

Categorical data are represented as observed frequencies and percentages. Continuous variables are presented as means and standard deviations. Statistical analyses were performed using SPSS Statistics 17.0 (SPSS Inc., Chicago, IL, USA). The IC group and NIC group were compared using independent *t* tests for continuous variables and chi-squared tests for categorical variables. Potential risk factors for IC (age, female gender, BMI, nail fit ratio, reduction technique, and greater trochanter nail entry) were analyzed using univariate and multivariate logistic regression. The same risk factors stated previously, along with IC occurrence, were also analyzed as potential risk factors for nonunion at 12 and 24 months after surgery using multivariate logistic regression. Differences were considered statistically significant at *p* < 0.05.

## Results

**Fig. 3:**
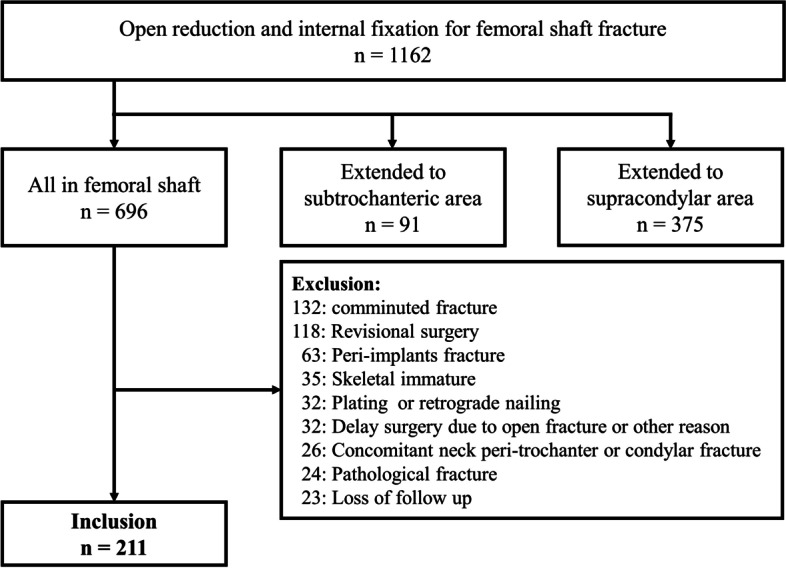
Flowchart depicting the inclusion and exclusion criteria and the number of patients enrolled in this study

Data on 1162 patients with femur fractures were retrospectively reviewed, and 696 patients had femoral shaft fractures. Of these patients, 485 patients were excluded according to the exclusion criteria described previously. The remaining 211 patients with simple femoral shaft fractures treated with antegrade interlocking nails were enrolled (Fig. 3). The study group comprised 123 men (58.3%) and 88 women (41.7%), and the average age was 31.8 years. The IC group comprised 44 patients, whereas the NIC group comprised 167 patients. The overall incidence of IC was 20.9%.

### Characteristics of iatrogenic fracture comminution

The characteristics of the IC are presented in Table 1. The most common location of ICs was the isthmus (59.1%), followed by the infraisthmic (29.5%) and supraisthmic (11.4%) areas. However, ICs located in infraisthmic areas accounted for the lowest union rates at 12 months (46.2%) and 24 months (69.2%) after surgery. Furthermore, most ICs occurred on the medial side (68.2%), whereas 13.6% occurred on the lateral side and 9.1% occurred on the anterior side. Three (6.8%) of our patients sustained long spiral or multiple direction fracture comminutions; these patients had the lowest union rates at 12 months (0%) and 24 months (66.7%) after surgery. Most ICs extended to the proximal side (59.1%), whereas 24.1% extended to the distal side and 6.8% extended in both directions. However, ICs that extended in both directions had the lowest union rates at 12 months (33.3%) and 24 months (66.7%) after surgery.

**Table 1:** Characteristics of iatrogenic fracture comminution

	Number (*n* = 44)	Union Within 12 months	Union rate 12 months	Union Within 24 months	Union rate 24 months
Iatrogenic comminution location					
Supra-isthmus	5 (11.4%)	5	100%	5	100%
Isthmus	26 (59.1%)	19	73.1%	22	84.6%
Infra-isthmus	13 (29.5%)	6	46.2%	9	69.2%
Iatrogenic comminution side					
Medial	30 (68.2%)	22	73.3%	24	80.0%
Lateral	6 (13.6%)	5	83.3%	6	100%
Anterior	4 (9.1%)	3	75.0%	3	75.0%
Posterior	1 (2.3%)	0	0%	1	100%
Long spiral or multiple fragments	3 (6.8%)	0	0%	2	66.7%
Iatrogenic comminution direction					
Proximal	26 (59.1%)	20	76.9%	22	84.6%
Distal	15 (24.1%)	9	60.0%	12	80.0%
Both direction	3 (6.8%)	1	33.3%	2	66.7%
Total	44	30	68.2%	36	81.8%

### Demographics and nonunion rates in the IC group and NIC group

**Fig. 4:**
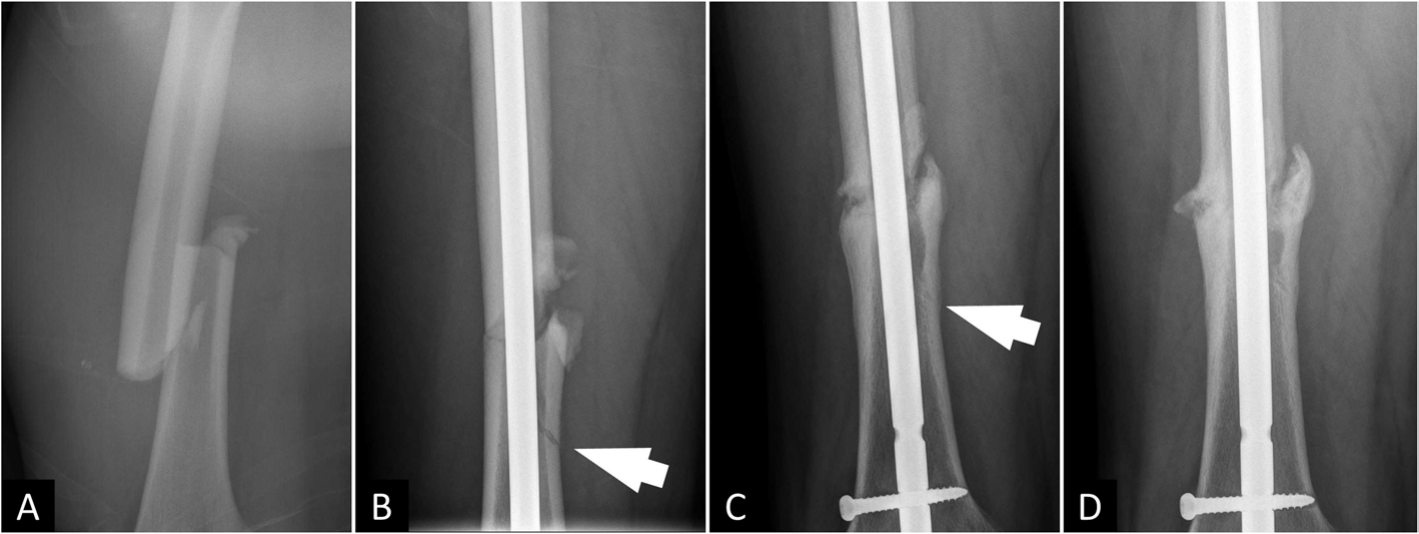
**A** A case of a 34-year-old man who suffered from right femoral shaft fracture after motor vehicle accident. **B** Iatrogenic fracture comminution over medial cortex which extended distally was noticed after surgery of open reduction and internal fixation. **C** At 14 months postoperatively, the fragment of iatrogenic comminution healed. However, the main fracture site remained nonunion. **D** The proximal locking screw was removed for dynamization at 17 months postoperatively. Partial union was noticed 6 months after dynamization

The demographic characteristics of age, gender, fracture side (right or left), BMI, fracture location, femur canal width, nail size, nail fit ratio, operative settings, open or closed reduction technique, and nail entry point were similar between the two study groups, as shown in Table 2. However, the nonunion rates at 12 and 24 months after surgery were significantly higher in the IC group than in the NIC group (12 months: 31.8% vs. 12.5%, *p* = 0.002; 24 months: 18% vs. 6.6%, *p* = 0.017). In the subgroup of patients who underwent open reduction (87 patients), the nonunion rate was significantly higher in patients who simultaneously developed IC than the patients without(60% vs. 20%, *p* = 0.002). Interestingly, on the plain film of all cases with nonunion, the iatrogenic comminution fragment healed with the main fragment despite that the initial fracture site remained nonunion, as shown in Fig. 4.

**Table 2:** Patient characteristics and nonunion in each study group

	Iatrogenic comminution (IC group) (*n* = 44)	None iatrogenic comminution (NIC group) (*n* = 167)	*p* value
Age	36.3 ± 19.2	30.6 ± 18.0	0.067
Gender			0.531
Male	24	99	
Female	20	68	
Side			0.288
Right	20	91	
Left	24	76	
BMI	23.53 ± 3.88	23.52 ± 4.42	0.988
Fracture location			0.077
Supraisthmius	5	8	
Isthmus	26	83	
Infraisthmus	13	76	
Canal width	9.97 ± 2.00	10.24 ± 1.79	0.391
Nail size	11.09 ± 1.24	11.04 ± 1.07	0.795
Nail fit ratio	1.14 ± 0.17	1.10 ± 0.15	0.131
Operative setting			0.949
Supine on fracture table	20	75	
Lateral decubitus	24	92	
Reduction technique			0.076
Open reduction	13	74	
Close reduction	31	93	
Nail entry point			0.231
Piriformis fossa	37	151	
Greater trochanter	7	16	
Nonunion with 12 months	14 (31.8%)	21 (12.5%)	0.002
Nonunion with 24 months	8 (18%)	11 (6.6%)	0.017

### Odds Ratios(OR) for predictors of iatrogenic fracture comminution

The results of the univariate and multivariate analysis of IC are shown in Table 3. In univariate analysis, age older than 35 years old significantly increased the odds ratio of IC occurrence (*p* = 0.03). In multivariate analysis, none of the potential predictors (age, gender, BMI, nail fit ratio, close reduction technique, and greater trochanter nail entry) were correlated with the occurrence of IC.

**Table 3:** Univariate and multivariate odds ratios of risk factors for the prediction of iatrogenic fracture comminution

Variable	Univariate analysis		Multivariate analysis	
	Odds ratio	*p*-value	Odds ratio	*p*-value
Age (≥ 35 years old)	2.13 (CI 1.07–4.23)	0.03	1.82 (CI 0.88–3.80)	0.11
Female	1.21 (CI 0.62–2.37)	0.57	1.06 (CI 0.51–2.21)	0.87
BMI (Every 1 kg/m^2^ increasing)	1.00 (CI 0.92–1.08)	0.95	0.99 (CI 0.92–1.08)	0.89
Nail fit ratio (Every 0.1 increasing)	1.17 (CI 0.95–1.44)	0.13	1.19 (CI 0.95–1.49)	0.13
Close reduction	1.90 (CI 0.93–3.88)	0.08	1.83 (CI 0.85–3.95)	0.12
GT entry nail	1.79 (CI 0.69–4.66)	0.24	1.66 (CI 0.60–4.58)	0.33

*CI* 95% confidence interval, *BMI* Body mass index, *GT* greater trochanter

### Odds ratios for predictors of nonunion at 12 and 24 months after surgery

The results of the multivariate analysis of nonunion at 12 and 24 months after surgery are shown in Tables 4 and 5. Among the variables included in the model, open reduction technique (12-month OR: 3.56, 95% CI: 1.48–8.55, *p* = 0.005; 24-month OR: 6.25, 95% CI: 1.89–20.41, *p* = 0.003), IC occurrence(12-month OR: 4.73, 95% CI: 1.85–12.10, *p* = 0.001; 24-month OR: 5.24, 95% CI: 1.57–17.49, *p* = 0.007) and higher BMI(12-month OR: 1.10, 95% CI: 1.00–1.20, *p* = 0.05; 24-month OR: 1.18, 95% CI: 1.05–1.32, *p* = 0.004) significantly increased the OR of nonunion. The coexistence of open reduction and IC occurrence significantly increased the odds ratio of fracture nonunion at 12-month(OR:10.13, 95% confidence interval(CI): 3.09–33.27, *p* = 0.0001) and 24-month postoperatively. (OR: 5.42, 95% CI: 1.49 to 19.70; *p* = 0.01).

**Table 4:** Odds ratios for prediction of nonunion at 12 months by multivariate logistic regression

Variable	Odds ratio	*p*-value
Age (≥ 35 years old)	0.99 (CI 0.41–2.40)	0.98
Female	1.29 (CI 0.54–3.06)	0.56
**BMI (Every 1 kg/m**^**2**^ **increasing)**	**1.10 (CI 1.00–1.20)**	**0.05**
Nail fit ratio (Every 0.1 increasing)	0.91 (CI 0.69–1.20)	0.52
**Open reduction**	**3.56 (CI 1.48–8.55)**	**0.005**
GT entry nail	2.13 (CI 0.70–6.51)	0.18
**Iatrogenic comminution**	**4.73 (CI 1.85–12.10)**	**0.001**

*CI* 95% confidence interval, *BMI* Body mass index, *GT* greater trochanter

**Table 5:** Odds ratios for prediction of nonunion at 24 months by multivariate logistic regression

	Odds ratio	*p*-value
Age (≥ 35 years old)	0.81 (CI 0.26–2.50)	0.71
Female	1.42 (CI 0.47–4.33)	0.54
**BMI (Every 1 kg/m**^**2**^ **increasing)**	**1.18 (CI 1.05–1.32)**	**0.004**
Nail fit ratio (Every 0.1 increasing)	1.08 (CI 0.75–1.55)	0.69
**Open reduction**	**6.23 (CI 1.89–20.55)**	**0.003**
GT entry nail	1.45 (CI 0.35–6.12)	0.61
**Iatrogenic comminution**	**5.24 (CI 1.57–17.49)**	**0.007**

*CI* 95% confidence interval, *BMI* Body mass index, *GT* greater trochanter

## Discussion

IMN is currently the gold standard treatment for femoral shaft fractures. However, nonunion of femoral shaft fractures represents a functional and economic burden for patients and presents a challenge for orthopedic surgeons. Many studies have investigated the possible risk factors related to nonunion in femoral shaft fractures following IMN [9–11]. However, little is known about the influence of the complication of IC on nonunion. In this retrospective analysis, we investigated the IC incidence, characteristics, and influence on fracture union following antegrade interlocking nailing of simple femoral shaft fractures. A total of 211 patients over 8 years were retrospectively analyzed. The overall incidence of IC was 20.9%. Approximately 60% of ICs occurred at the level of the isthmus, and almost 70% were located on the medial side. ICs with a long spiral structure or multidirectional extension accounted for the highest nonunion rate. The IC group exhibited a significantly higher nonunion rate at both 12 months and 24 months after surgery than did the non-IC group. Multivariate analysis also demonstrated that open reduction technique, IC occurrence and higher BMI significantly increased the OR of nonunion at both 12 months and 24 months after surgery.

In our study, most ICs occurred at the level of the isthmus and involved the medial cortex. Possible reasons for IC included oversized cortical reaming, rotation of the nail during insertion, and a medially directed insertion angle during nailing. When a rigid femoral nail is being introduced through a lateral entry portal, the distal tip may impact the medial femoral cortex while the proximal portion of the nail is not fully engaged. The optimal entry point for antegrade nailing of the femur to minimize the risk of iatrogenic fracture and malreduction is debated [12–14]. Prasarn et al. conducted a retrospective study of 227 patients with femoral shaft fractures treated with femoral nails using lateral entry [15]. Iatrogenic fractures were found in 16 patients (7%), whereas IC was noted in 7 patients (3%). The authors analyzed the lateral radiographs and concluded that the anterior entry portal significantly increased the risk of iatrogenic fracture compared with a middle entry portal (13% vs. 0%, *p* = 0.001). Tupis et al. conducted a finite element analysis and demonstrated higher strain levels in the peritrochanteric region when trochanteric entry was used [16]. However, the strain levels in the diaphysis were similar. Current evidence is insufficient to support a correlation between different entry points and the occurrence of IC. We proposed several potential factors that may be associated with IC, including older age, female sex, higher BMI, a higher nail fit ratio, open reduction technique, and greater trochanter nail entry. In univariate analysis, older age significantly correlated with the occurrence of IC. Ageing was proved to be positively correlated with dynamically deforming of the femoral shaft [17]. These morphological changes may cause a mismatch between the femoral bow and the curvature of the intramedullary nail [18]. Although the result of the multivariate analysis failed to demonstrate the correlation between occurrence of IC and aforementioned factors, we believed that ageing related morphological changes potentially affected intramedullary nail insertion. It appears that a less straight of current nail designs is warranted.

The most important finding of the present study was that IC significantly increased the nonunion rate in simple femoral shaft fractures at 12 and 24 months after surgery. Studies have demonstrated that a greater fracture severity was associated with a higher nonunion rate [19, 20]. Noumi et al. conducted a retrospective analysis involving 89 femoral shaft open fractures treated with IMN [21]. The nonunion rates for AO/OTA type A, B, and C fractures were 6.1, 16.7, and 41.7%, respectively (*p* = 0.014). Multivariate analysis revealed that the fracture grade according to AO classification was significantly correlated with the occurrence of nonunion. Hamahashi et al. conducted a retrospective analysis involving 51 patients with femoral shaft fractures with third fragments treated with IMN [20]. The overall incidence of delayed union was 33.3%, and displacement of the third fragment was the only risk factor identified for delayed union. On the other hand, patients with iatrogenic comminution delayed in the progress of weight-bearing. Brumback et al. revealed that immediate weight-bearing after intramedullary nailing for femoral shaft fracture reduced the need for prolonged rehabilitation [22]. Delayed weight-bearing was identified as independent predictor of nonunion after intramedullary nailing of femoral shaft fractures [11]. Therefore, we believed that IC contributed to greater fracture severity and delayed the timing for weightbearing, and thus negatively influenced the process of fracture union. Therefore, extreme caution should be taken when introducing IMN to avoid this complication.

The nonunion of femoral shaft fractures is complex and multifactorial. Many studies have investigated the potential risk factors for nonunion [10, 11, 21]. In general, the factors identified can be classified into four categories: patient-related factors, environment factors, injury-related factors, and surgery-related factors. Wu et al. reported that fracture site comminution; fracture at the proximal third junction; and comorbidities such as obesity, hypertension, and diabetes mellitus were associated with a higher risk of nonunion [23]. A systematic review conducted by Santolini et al. highlighted 10 risk factors for long bone fracture nonunion [24]. Surgery-related factors, including open reduction technique, presence of a postoperative fracture gap, and mechanical instability, were mentioned. Injury-related factors, including open fracture, wedge or comminuted fractures, and a high degree of initial fracture displacement, also played a critical role in fracture nonunion [24]. Our study confirmed previous findings that an open reduction technique and obesity were associated with fracture nonunion at 12 and 24 months after surgery. Furthermore, IC was a major factor in the development of fracture nonunion. The coexistence of open reduction and IC occurrence also significantly increased the risk of fracture nonunion at 12-month and 24-month postoperatively.

Several limitations should be acknowledged. First, the data were collected retrospectively from a single trauma center. We were not able to obtain information on the timing of iatrogenic fracture comminution, and thus, we could not determine whether the fracture comminution occurred during fracture reduction, canal reaming, or nail insertion. Second, the fracture was evaluated preoperatively on plain film only. Since computed tomography(CT) scan was not a routine examination for femoral shaft fracture, a more complex fracture might be mistaken for simple fracture without the confirmation of preoperative CT scan. Third, the sample size was relatively small, and the study may have lacked sufficient power to detect meaningful differences in union rates and the incidence of other complications between the two groups. Fourth, we reported a 1-year union rate of 83%, which is lower than the rates provided in previous studies [3, 20]. We hypothesize that this phenomenon could be related to the frequent use of the open reduction technique (41%) among our study population. However, the 2-year union rate in our study population was 94%, which is comparable with the union rates reported in the literature [3, 20].

## Conclusion

IC was non-rare in simple femoral shaft fracture with incidence of 20.9% in our cohort. IC mostly occurred at the level of the isthmus, frequently involved with the medial cortex, and significantly increased nonunion rates at 12 and 24 months after surgery. Age older than 35 years old showed a trend toward increasing risk of IC, while gender, BMI, nail fit ratio, close reduction technique, and greater trochanter nail entry were not associated with IC occurrence.

## Data Availability

All data generated or analyzed during this study are included in this published article.

## Abbreviations

IC: Iatrogenic comminution
BMI: Body mass index
IMN: Intramedullary nailing
OR: Odds ratios
CI: Confidence interval
CT: Computed tomography
